# Entropic Dynamics Approach to Relational Quantum Mechanics

**DOI:** 10.3390/e27080797

**Published:** 2025-07-26

**Authors:** Ariel Caticha, Hassaan Saleem

**Affiliations:** Physics Department, University at Albany-SUNY, Albany, NY 12222, USA

**Keywords:** Entropic Dynamics, relational Quantum Mechanics, foundations of Quantum Mechanics, generally covariant Quantum Mechanics, problem of time

## Abstract

The general framework of Entropic Dynamics (ED) is used to construct non-relativistic models of relational Quantum Mechanics from well-known inference principles—probability, entropy and information geometry. Although only partially relational—the absolute structures of simultaneity and Euclidean geometry are still retained—these models provide a useful testing ground for ideas that will prove useful in the context of more realistic relativistic theories. The fact that in ED the positions of particles have definite values, just as in classical mechanics, has allowed us to adapt to the quantum case some intuitions from Barbour and Bertotti’s classical framework. Here, however, we propose a new measure of the mismatch between successive states that is adapted to the information metric and the symplectic structures of the quantum phase space. We make explicit that ED is temporally relational and we construct non-relativistic quantum models that are spatially relational with respect to rigid translations and rotations. The ED approach settles the longstanding question of what form the constraints of a classical theory should take after quantization: the quantum constraints that express relationality are to be imposed on expectation values. To highlight the potential impact of these developments, the non-relativistic quantum model is parametrized into a generally covariant form and we show that the ED approach evades the analogue of what in quantum gravity has been called the problem of time.

## 1. Introduction

Many, perhaps all, the difficulties in the study of space and of time can be traced to their invisibility. We do not see space; we see matter in space. We do not see time; we see changes in things. Although these studies have resulted in an increased understanding of the properties of matter in space–time the problem remains that it is not always clear how to disentangle which properties should be attributed to matter and which to space. Newton managed to evade the issue and created a successful science of mechanics but only at the cost of adopting an absolute concept of motion relative to an abstract “mathematical” space and time. Leibniz objected that such absolute positions would not be observable and therefore not real. Instead, he advocated for a concept of motion defined in terms of the observable relative distances. Newton had, however, a definite dynamical theory to back him up, while Leibniz had no such support. As a result Newton won the day, and the relational argument lay largely dormant for almost two centuries until revived by Mach and specially by Einstein. In the latter decades of the 20th century, as a stepping stone towards a quantum theory of gravity, the thrust towards a fully relational dynamics was once again resumed in the form of Hamiltonian formulations of general relativity [1,2,3,4]. More recently, the understanding of relational motion achieved a certain level of completion—at least within the context of classical physics—with the explicitly Machian line of research that originates with J. Barbour and B. Bertotti [5,6,7].

Barbour and Bertotti’s basic insight [5] was that a formulation in terms of the relative interparticle distances is not practical. Instead, one should focus attention on the fact that two configurations that differ by arbitrary rigid (i.e., global) displacements and/or rigid rotations describe exactly the same physical situation. Thus, in formulating a relational dynamics, whatever measure one adopts to quantify the change from one configuration to the next, the actual intrinsic change should remain unaffected by independent rigid shifts and rotations of each configuration. This is achieved through a technique Barbour and Bertotti called “best matching” (BM). The idea is to introduce a quantitative measure of the “mismatch” between two successive configurations and then shift and rotate one configuration *relative* to the other to find the location that minimizes the mismatch. Thus, motion is not defined as relative to an absolute space, but relative to the earlier state of the system itself. Barbour and Bertotti’s intrinsic change and best matching have added an interesting twist: what is relational is not the notion of space but the notion of change.

With Quantum Mechanics (QM), however, new problems arise. In its standard Copenhagen interpretation QM is manifestly non-relational. It lives in Newton’s absolute space and time, or at best, in Minkowski’s absolute space–time, and its particles do not have definite positions, much less definite relative distances. But then the interpretations of QM have always been a source of controversy and many have been proposed. One approach inspired by Barbour and Bertotti is due to Gryb [8]. Another relevant example, Rovelli’s relational interpretation, is tailored to formulating a background-independent quantum theory of gravity [9,10]. We shall follow a different path. First, however, we must clarify a potential confusion between this line of research and the different line of research by the same name pursued by Rovelli and collaborators (see [9,11]). As we pointed out above, we use the term “relational” in a sense that can be traced historically in the 18th century back to Newton and Leibniz and the Leibniz–Clarke correspondence. In later times, it can be traced through Mach, Einstein, and finally to the modern Barbour–Bertotti version. Our contribution is to provide a quantum version of this historical evolution. It is this history that extends through several centuries that justifies our use of the name ‘Relational Quantum Mechanics’ as appropriate. The name has also been adopted by Rovelli and collaborators in the very different context of quantum measurement and of correlations among observables. Their line of research has little to do with the subject of this paper.

Our goal here is to formulate a non-relativistic relational QM within the framework of Entropic Dynamics (ED) [12,13]. Entropic Dynamics is a subject within the subfield of theoretical physics that is currently called Foundations of Physics. Its goal is to derive or “reconstruct” the standard formalism of QM and to resolve the longstanding conceptual problems (wave–particle duality, the measurement problem, the ontic vs. the epistemic interpretation of the wave function, wave function collapse, etc.) that have plagued QM since its origin.

The mathematical formalism is derived using well-established tools and principles of inference—probabilities, entropies, and information geometry. (For a pedagogical review see [14]). With regard to interpretation, one appealing feature is that ED achieves a clear separation between the epistemic and the ontic elements. This allows ED to solve the measurement problem while evading the no-go theorems that afflict other realist ψ-epistemic interpretations [15]. ED is a conservative theory in that it grants a definite ontic status to things such as the positions of particles—they have definite values at all times—and grants a definite epistemic status to probabilities and wave functions without invoking exotic quantum probabilities. In contrast, ED is radically non-classical in that there is no ontic dynamics; ED is a purely epistemic dynamics of probabilities.

As a method of quantization, ED is singled out in that it does not rely on the prior formulation of a classical dynamics to which one must append some ad hoc quantization rules; therefore, there are no operator-ordering ambiguities, because there are no operators. In the present context, one does not first formulate a classical Machian dynamics à la Barbour–Bertotti with a corresponding classical criterion for best matching. Instead, one directly formulates a quantum theory with its corresponding quantum best matching criterion (BM) based on the natural geometrical tools available to ED, namely, information and symplectic geometry which lead, eventually, to the Hilbert space inner product.

Some of Barbour and Bertotti’s ideas can be readily adapted and imported into the ED framework. In an earlier work towards a relational ED [16], some of the conceptual issues were successfully settled: in a dynamics of probabilities, it should come as no surprise that the configurations to be compared involve probability distributions. A second crucial question is the particular choice of mismatch measure. In the classical context, the measure adopted by Barbour and Bertotti was borrowed from Jacobi’s classical action principle and amounts to a variation on a least-squares mismatch [5]. In [16], a mismatch measure based on information geometry was adopted which was natural in the probabilistic context but, unfortunately, that measure proved less than satisfactory in practice. In the meantime, further formal developments in the ED reconstruction of QM [13] have shown that the geometrical structures relevant to QM involve not just information geometry but also symplectic geometry. Here, we deploy the recent development of ED as Hamilton–Killing flows and propose a quantum BM criterion to derive a relational QM of particles.

Our present concern with a non-relativistic relational QM implies that the resulting theory will necessarily exhibit some still provisional and therefore unsatisfactory features. Thus, what we produce here are toy models that will allow us to ask questions and test ideas that will prove useful in the construction of more realistic relativistic theories.

One such question is what we mean by “relational”. The idea behind absolute space and time is that these structures exist independently of matter. In contrast, in a fully relational approach all assertions about space and time are to be ultimately interpreted as assertions about relations involving matter and the relevant probabilities and wave functions.

Another question is about the nature of those relations: with respect to what transformations is the theory supposed to be relational? In a more realistic model that includes quantum fields and gravity, one might expect the transformations in question to include local diffeomorphisms. Here, in a non-relativistic setting, we shall be more modest and include only rigid translations and rotations. Our models will, therefore, be only partially relational; they will retain some absolute structures including absolute simultaneity and the Euclidean geometry of space.

We will argue that as previously formulated ED is already temporally relational. One goal is to make this feature more explicit and, having done so, to formulate a non-relativistic quantum model that is generally covariant with respect to time. This toy model exemplifies a strategy that successfully evades the notorious “problem of time” in quantum gravity [17,18]. Briefly, the “problem of time” arises in the canonical quantization of gravity because in any generally covariant theory the physical quantum states are constrained to be annihilated by the quantum Hamiltonian, $\hat{H}\Psi =0$. Thus, the analogue of the Schrödinger equation—the Wheeler–DeWitt equation—implies that physical states cannot evolve and that nothing ever happens.

Here, once again, we wish to avoid the misunderstandings that might arise from confusion with another approach to dynamics due to Page and Wootters [19] who proposed a *timeless picture of quantum dynamics*. Their approach to time is instrumentalist: time is what is measured by a clock. Their universe consists of two systems, one being the system of interest and the other a clock, and it is assumed to be in a stationary state—thus, the *timeless* picture. Dynamics consists of tracking the correlations due to entanglement between the system and the clock. (For a pedagogical treatment with more recent references, see [20]). As we shall see below, relational time in the ED approach differs in several crucial ways. For example, our universe is not necessarily in a stationary state and there are no external clocks. Furthermore, as one might expect in an *entropic* dynamics, there is a natural arrow of time, even though invariance under time reversal need not be violated.

In Section 2, as a preliminary to defining a spatially relational ED, we establish the subject matter—the ontic microstates—and define the concept of intrinsic change. The ED of intrinsic change is formulated in Section 3 where we emphasize that the notion of entropic time associated to ED is already relational in the sense that the clock that defines entropic time is the quantum system itself—there are neither external clocks nor an absolute external time. In Section 4, we formulate the quantum criterion for best matching using the natural geometric tools available to ED. In Section 4.1, we construct a QM model that is relational with respect to rigid translations, and then in Section 4.2 a QM model that is relational with respect to both rigid translations and rotations. In Section 5, we pursue formal developments: we write the relational QM models in terms of a Hamiltonian action principle. Then, in order to make the temporal relationality more explicit we rewrite our ED models in a form that is “generally covariant”. The procedure is well known from the canonical formulation of general relativity and results in a “parametrized ED” [21,22]. Our quantum toy model serves to illustrate how the ED approach evades the notorious problem of time that afflicts the canonical quantization of gravity. Finally, in Section 6 we summarize our conclusions.

## 2. Spatial Relationality

### 2.1. The Ontic Microstates

The system of interest consists of *N* particles that live in a three-dimensional flat space X with metric $\delta_{ab}$. The microstate of the *N* particle system is a point $x\in X_{N}$ in the 3N-dimensional ontic configuration space, $X_{N}=X⊗\cdots ⊗X$, with coordinates $x_{n}^{a}$ (a=1,2,3 is the spatial index and n=1…N labels the particle).

The two central assumptions concerning the ontology are that the particles have definite positions and that they follow continuous paths. (Generic paths will turn out to be non-differentiable). Our goal is to predict those positions on the basis of information that happens to be limited; therefore, the best we can hope to do is assign a probability distribution ρ(x) and study its time evolution. The continuity of the paths leads to an important simplification because it implies that the motion can be analyzed as a sequence of simple infinitesimally short steps. To find the probability $P(x^{\prime }|x)$ that the system takes a short step from $x_{n}^{a}$ to ${x^{\prime }}_{n}^{a}=x_{n}^{a}+\Delta x_{n}^{a}$, we use the method of maximum entropy. Then, these short steps will be iterated to find the evolving $\rho_{t}\left(x\right)$. So far, this is standard ED as described in [12,14].

### 2.2. Intrinsic Change

For a relational ED, we stipulate that the specification of the microstate in terms of the coordinates $x_{n}^{a}$ is redundant in the sense that shifting all particles by rigid (or global) translations or rotations does not lead to a different ontic state. Relationism about space and time can take different forms depending on the nature of the transformations being considered. Consider spatial transformations (displacements) of the form,

(1) $$\begin{array}{cc} \mathrm{ST}1 & :\;{\vec{x}}^{\prime }=R_{0}\;\vec{x}+{\vec{\lambda }}_{0}, \end{array}$$

(2) $$\begin{array}{cc} \mathrm{ST}2 & :\;{\vec{x}}^{\prime }=R_{0}\;\vec{x}+\vec{\lambda }\left(t\right), \end{array}$$

(3) $$\begin{array}{cc} \mathrm{ST}3 & :\;{\vec{x}}^{\prime }=R\left(t\right)\;\vec{x}+\vec{\lambda }\left(t\right). \end{array}$$

The idea is that with ST1 all particles are subject to rigid displacements ${\vec{\lambda }}_{0}$ and rotations $R_{0}$ that are independent of location and time. Such transformations are not meant to generate a different ontic state and they preserve the form of the equations of motion. They reflect a weak form of relationism in that the whole of space–time is rigidly shifted and rotated. In this paper, we shall be concerned with constructing QM models that reflect the stronger relationism implied by transformations of type ST2 and the even stronger ones implied by ST3.

Transformations ST2 allow greater freedom to displace space by different rigid amounts $\vec{\lambda }\left(t\right)$ at different instants. The corresponding ST2 model exhibits a fair relationality with respect to rigid translations but much less so with respect to rotations. As an aside, the subgroup of ST2 transformations with

(4) $$\mathrm{ST}4:\;{\vec{x}}^{\prime }=R_{0}\;\vec{x}+{\vec{\lambda }}_{0}+{\vec{v}}_{0}t,$$

where ${\vec{v}}_{0}$ and ${\vec{\lambda }}_{0}$ are constant vectors, describes Galilean relativity. Since the corresponding notion of relative space is what Newton described in the *Scholium* to the *Principia*, systems that are relational not with respect to the full ST2 transformations but only with respect to the more limited subgroup ST4 reflect a “Newtonian” relationism.

Transformations ST3 allow greater relationality with respect to rotations. Just as in classical mechanics, transforming to rotating frames of reference has dynamical consequences; total angular momentum is dynamically relevant. More on this later.

Let us then consider shifts of the form

(5) $$x_{n}^{a}\to x_{n}^{a}+\xi_{n}^{a}\;\mathrm{where}\;\xi_{n}^{a}\left(x_{n}\right)=\varepsilon^{abc}\zeta_{b}x_{nc}+\lambda^{a}$$

($\lambda^{a}$ and $\zeta^{a}$ are some arbitrary vectors independent of the particle *n* and of the position $x_{n}^{a}$ and $\varepsilon^{abc}$ is the Levi–Civita tensor). Since $x_{n}^{a}$ and $x_{n}^{a}+\xi_{n}^{a}$ represent the same initial state and ${x^{\prime }}_{n}^{a}$ and ${x^{\prime }}_{n}^{a}+{\xi^{\prime }}_{n}^{a}$ represent the same final state, the short step from one to the other will be represented by

(6) $$\left({x^{\prime }}_{n}^{a}+{\xi^{\prime }}_{n}^{a}\right)-\left(x_{n}^{a}+\xi_{n}^{a}\right)=\Delta x_{n}^{a}+\Delta \xi_{n}^{a},$$

The two configurations are said to be “best matched” when the two vectors $\Delta \lambda^{a}$ and $\Delta \zeta^{a}$ are chosen to minimize a certain measure of mismatch to be defined later. Finding the optimal $\Delta \lambda_{\mathrm{best}}^{a}$ and $\Delta \zeta_{\mathrm{best}}^{a}$ amounts to deciding which position ${x^{\prime }}^{a}$ at the later instant *is the same as* the position $x^{a}$ at the earlier instant. It provides a criterion of “equilocality” between successive instants. The quantity

(7) $$\hat{\Delta }x_{n}^{a}=\Delta x_{n}^{a}+\Delta \xi_{n\;\mathrm{best}}^{a}$$

will be called the *intrinsic change*. Two successive configurations are “intrinsically identical” when equilocality has been established and $\hat{\Delta }x_{n}^{a}=0$. At this point in our argument, the optimal $\Delta \lambda_{\mathrm{best}}^{a}$ and $\Delta \zeta_{\mathrm{best}}^{a}$ are not yet known. However, to proceed we will assume that equilocality has been established through some trial shift $\Delta \xi_{n\;\mathrm{best}}^{a}$ to be determined later. From now on, we drop the subscript “best” and write $\Delta \xi_{n\;\mathrm{best}}^{a}=\Delta \xi_{n}^{a}$.

## 3. The Entropic Dynamics of Intrinsic Change

Except for the replacement of the change $\Delta x_{n}^{a}$ by the intrinsic change $\hat{\Delta }x_{n}^{a}$, the contents of this section parallel closely the material described in [12] or in Chapter 11 of [14].

### 3.1. The Transition Probability for a Short Step

To find the transition probability, we maximize the entropy of $P\left(x^{\prime }|x\right)$ relative to a prior $Q\left(x^{\prime }|x\right)$,

(8) $$S\left[P,Q\right]=-\int dx^{\prime }P\left(x^{\prime }|x\right)\log\frac{P\left(x^{\prime }|x\right)}{Q\left(x^{\prime }|x\right)},$$

subject to constraints that codify the relevant physical information. We choose a prior $Q(x^{\prime }|x)$ that describes a state of knowledge that is common to all short steps *before* we take into account the additional information that is specific to each specific short step. We require it to incorporate the information that the particles take infinitesimally short steps but *Q* is otherwise maximally uninformative in the sense that it must reflect the translational and rotational invariance of the space X and expresses total ignorance about any correlations. (Such a prior can itself be derived from the principle of maximum entropy). The chosen prior is

(9) $$Q\left(x^{\prime }|x\right)\propto \exp\left(-\frac{1}{2}\sum_{n}\alpha_{n}\delta_{ab}\hat{\Delta }x_{n}^{a}\hat{\Delta }x_{n}^{b}\right).$$

The multipliers $\alpha_{n}$ are constants that may depend on the index *n* in order to describe non-identical particles. To enforce the fact that the steps are meant to be infinitesimally short, $\alpha_{n}$s will eventually be taken to infinity.

The information that induces the directionality and correlations specific to each individual short step is introduced by imposing one additional constraint,

(10) $$\sum_{n}\langle \hat{\Delta }x_{n}^{a}\rangle \frac{\partial \varphi \left(x\right)}{\partial x_{n}^{a}}=\int dx^{\prime }P\left(x^{\prime }|x\right)\sum_{n}\hat{\Delta }x_{n}^{a}\frac{\partial \varphi \left(x\right)}{\partial x_{n}^{a}}=\kappa^{\prime }.$$

The function $\varphi \left(x\right)=\varphi \left(x_{1},\ldots ,x_{N}\right)$, called the “drift” potential, will play a central role in what follows. The quantity $\kappa^{\prime }$ is a small but for now unspecified constant. Maximizing the entropy (8) subject to the constraints (10) and normalization leads to a Gaussian distribution,

(11) $$P\left(x^{\prime }|x\right)=\frac{1}{Z}\exp\left[-\sum_{n}\frac{\alpha_{n}}{2}\delta_{ab}\left(\hat{\Delta }x_{n}^{a}-\frac{\alpha^{\prime }}{\alpha_{n}}\partial_{n}^{a}\varphi \right)\left(\hat{\Delta }x_{n}^{b}-\frac{\alpha^{\prime }}{\alpha_{n}}\partial_{n}^{b}\varphi \right)\right].$$

where *Z* is a normalization constant, $\left\{\alpha_{n},\alpha^{\prime }\right\}$ are Lagrange multipliers, and $\partial_{na}=\partial /\partial x_{n}^{a}$. Substituting (7) into (11), a generic displacement $\Delta x_{n}^{a}={x^{\prime }}_{n}^{a}-x_{n}^{a}$ can then be written as the sum of an expected drift plus a fluctuation,

(12) $$\Delta x_{n}^{a}=\langle \Delta x_{n}^{a}\rangle +\Delta w_{n}^{a},$$

where

(13) $$\langle \hat{\Delta }x_{n}^{a}\rangle =\frac{\alpha^{\prime }}{\alpha_{n}}\delta^{ab}\partial_{nb}\varphi ,\;\langle \Delta x_{n}^{a}\rangle =\frac{\alpha^{\prime }}{\alpha_{n}}\delta^{ab}\partial_{nb}\varphi -\Delta \xi_{n}^{a},$$

(14) $$\langle \Delta w_{n}^{a}\rangle =\langle \hat{\Delta }x_{n}^{a}-\frac{\alpha^{\prime }}{\alpha_{n}}\partial_{n}^{a}\varphi \rangle =0,\;\mathrm{and}\;\langle \Delta w_{n}^{a}\Delta w_{n^{\prime }}^{b}\rangle =\frac{1}{\alpha_{n}}\delta^{ab}\delta_{nn^{\prime }}.$$

### 3.2. Temporal Relationality: Entropic Time

An epistemic dynamics of probabilities demands an epistemic notion of time. The construction of time involves introducing the concept of an instant, verifying that the instants are suitably ordered, and adopting a convenient definition of duration, that is, a measure of the interval between instants. The construction is intimately related to information and inference.

An instant is an epistemically complete state specified by information—codified into the functions $\rho_{t}\left(x\right)$ and $\varphi_{t}\left(x\right)$—that is sufficient for generating the next instant. Thus, the instant we call the present is defined so that, given the information codified into the present instant, the future is independent of the past. Formally, ED is a Markovian process: if the distribution $\rho_{t}\left(x\right)$ and the drift potential $\varphi_{t}\left(x\right)$ refer to the instant *t*, then the distribution

(15) $$\rho_{t^{\prime }}\left(x^{\prime }\right)=\int dx\;P\left(x^{\prime }|x\right)\rho_{t}\left(x\right)$$

generated by $\rho_{t}\left(x\right)$ and $P\left(x^{\prime }|x\right)$ defines what we mean by the “next” instant $t^{\prime }$. (This takes care of the equation of evolution for $\rho_{t}\left(x\right)$; below, we will return to specifying the evolution of $\varphi_{t}\left(x\right)$).

In ED, time is *constructed* instant by instant and since no reference is made to external clocks the dynamics does not unfold in a pre-existing externally given absolute time or space–time. Furthermore, the construction leads to a sequence of instants that are ordered because the transition probability $P\left(x^{\prime }|x\right)$ is determined by *maximizing* an entropy and, by its very construction, there is a natural arrow of time.

The last ingredient—duration, the interval Δt between successive instants—is defined to simplify the dynamics. The description is simplest when it reflects the symmetry of translations in space and time that are typical of the weak interactions that characterize non-relativistic physics. In Newtonian mechanics, the prototype of a clock is a free particle that moves equal distances in equal times. In ED, the dynamics is described by $P\left(x^{\prime }|x\right)$ and we define duration so that the multipliers $\alpha_{n}$ and $\alpha^{\prime }$ are constants independent of *x* and *t* so they lead to an entropic time that resembles a (relational) Newtonian time in that it flows “equably everywhere and everywhen.” Here too, we define duration so that for sufficiently short steps there is a well-defined drift velocity. As we see from Equation (13) this is achieved by setting the ratio $\alpha^{\prime }/\alpha_{n}\;$proportional to Δt. Thus, *the transition probability provides us with a clock*. For future convenience, the proportionality constants will be expressed in terms of some particle-specific constants $m_{n}$,

(16) $$\frac{\alpha^{\prime }}{\alpha_{n}}=\frac{1}{m_{n}}\Delta t.$$

At this point, the constants $m_{n}$ receive no interpretation beyond the fact that their dependence on the particle label *n* recognizes that the particles need not be identical but later we shall see that $m_{n}$s will be identified with the particle masses. To explore the consequences of the choice (16), we assume $\alpha^{\prime }=$ 1/η is a constant and choose η so that if Δt has units of time, then $m_{n}$ has units of mass. Then,

(17) $$\alpha^{\prime }=\frac{1}{\eta }\;\mathrm{so}\;\mathrm{that}\;\alpha_{n}=\frac{m_{n}}{\eta \Delta t}.$$

We rewrite the generic short step Equations (12)–(14) in a more streamlined notation,

(18) $$\Delta x^{A}=\hat{\Delta }x^{A}-\Delta \xi^{A}=\langle \Delta x^{A}\rangle +\Delta w^{A},$$

where A=(n,a) is a composite index that includes both the particle *n* and the spatial index *a* so that $x_{n}^{a}=x^{A}$. To simplify, we write the spatial shift in configuration space as

(19) $$\xi^{A}\left(x\right)=\xi_{n}^{a}\left(x_{n}\right).$$

We also introduce the mass tensor,

(20) $$m_{AB}=m_{n}\delta_{AB}=m_{n}\delta_{nn^{\prime }}\delta_{ab}\;\mathrm{and}\;\mathrm{its}\;\mathrm{inverse}\;m^{AB}=\frac{1}{m_{n}}\delta^{AB}.$$

Then, we find that the shift $\Delta \xi^{A}$ affects the expected steps,

(21) $$\langle \Delta x^{A}\rangle =\langle \hat{\Delta }x^{A}\rangle -\langle \Delta \xi^{A}\rangle =\Delta t\;m^{AB}\partial_{B}\varphi -\Delta \xi^{A},$$

but, of course, does not affect the fluctuations,

(22) $$\langle \Delta x^{A}\Delta x^{B}\rangle =\langle \hat{\Delta }x^{A}\hat{\Delta }x^{B}\rangle +O\left(\Delta t^{2}\right).$$

Therefore,

(23) $$\hat{\Delta }w^{A}=\Delta w^{A}\;\mathrm{with}\;\langle \Delta w^{A}\rangle =0\;\mathrm{and}\;\langle \Delta w^{A}\Delta w^{B}\rangle =\eta \Delta t\;m^{AB},$$

which remain large $\Delta w∼O(\Delta t^{1/2})$ and essentially isotropic. This leads us to expect that the optimal shift Δξ is of the order Δt so that Δξ≪Δw.

We conclude this section noting that the entropic time we have constructed here is already fully relational. Time is the sequence of instants that are epistemically complete: each instant is defined by information codified into the distributions $\rho_{t}\left(x\right)$ and $\varphi_{t}\left(x\right)$ that serve to define the next instant. The evolution Equation (15) is used both to define the dynamics and to construct time itself instant by instant. There is neither an absolute time nor are there external clocks. As we can see from (21) or (23), the system is its own clock in that the duration Δt can be defined either from the expected intrinsic change $\langle \hat{\Delta }x^{A}\rangle$ or from the fluctuations $\langle \Delta w^{A}\Delta w^{B}\rangle$.

### 3.3. The Probability-Evolution Equation

The dynamical equation of evolution, Equation (15), can be written in differential form as a continuity equation (a Fokker–Planck equation),

(24) $$\partial_{t}\rho \left(x,t\right)=-\partial_{A}\left[\rho \left(x,t\right)v^{A}\left(x,t\right)\right],$$

where $v^{A}$ is the velocity of the probability flow, or current velocity,

(25) $$v^{A}\left(x,t\right)=m^{AB}\partial_{B}\phi \left(x,t\right)-{\dot{\xi }}^{A}\;\mathrm{with}\;\phi =\varphi -\eta \log\rho^{1/2},$$

and ${\dot{\xi }}^{A}=\Delta \xi^{A}/\Delta t$. The derivation, which involves a technique that is well known from diffusion theory [23], follows the same steps as the analogous (non-relational) derivation found in [14].

It turns out that the crucial drift potential φ that was introduced via the maximum entropy constraint (10) will always appear in the particular combination of φ and logρ given in (25). This justifies introducing the function ϕ(x,t) that will play three separate roles: first, as we saw above, it is related to a constraint in the maximization of entropy; second, if the probabilities ρ(x,t) are considered as generalized coordinates, then ϕ(x,t) turns out to be the momentum that is canonically conjugate to them; and third, ϕ(x,t) is the phase of the quantum wave function, $\psi =\rho^{1/2}e^{i\phi /\hbar }$.

The current velocity $v^{A}$ receives three types of contributions. The first two are the familiar drift and osmotic velocities described through the gradient of the “phase” ϕ [14]. The third contribution is the term that implements relationality, the shift velocity ${\dot{\xi }}^{A}$.

We now return to the continuity Equation (24) and rewrite it in an alternative form. The important observation is that a functional $\tilde{H}\left[\rho ,\phi \right]$ can be found such that (24) can be written as

(26) $$\partial_{t}\rho_{t}\left(x\right)=\frac{\delta \tilde{H}}{\delta \phi (x)}.$$

The desired $\tilde{H}$ satisfies

(27) $$-\partial_{A}\left[\rho_{t}m^{AB}\left(\partial_{B}\phi -{\dot{\xi }}_{B}\right)\right]=\frac{\delta \tilde{H}}{\delta \phi (x)}\;\mathrm{where}\;{\dot{\xi }}_{B}=m_{BC}{\dot{\xi }}^{C},$$

which is a linear functional equation that can be easily integrated. The result is

(28) $$\tilde{H}\left[\rho ,\phi \right]=\int dx\;\frac{1}{2}\rho m^{AB}\left(\partial_{A}\phi -{\dot{\xi }}_{A}\right)\left(\partial_{B}\phi -{\dot{\xi }}_{B}\right)+\tilde{F}\left[\rho \right],$$

where the unspecified functional $\tilde{F}\left[\rho \right]$ is an integration constant (i.e., independent of ϕ). This maneuver has allowed us to identify the kinetic part of the ED Hamiltonian and a “potential” $\tilde{F}$ that is independent of ϕ but could potentially also depend on *x* and on *t*. More importantly, it suggests a Hamiltonian framework in which the epistemic variables ρ and ϕ are canonically conjugate. The epistemic configuration space (or “e-configuration” space) is the simplex,

(29) $$S=\left\{\rho |\;\rho (x)\geq 0\;;\;\int dx\;\rho (x)=1\right\}.$$

The epistemic phase space (or “e-phase” space) is the cotangent bundle $T^{*}S$ [14]. As is well known, dealing with normalized probabilities is a technical inconvenience that can be handled by discarding the normalization constraint and dealing instead with the space

(30) $$S^{+}=\left\{\rho |\;\rho (x)\geq 0\right\}$$

of unnormalized probabilities and its associated cotangent bundle $T^{*}S^{+}$.

### 3.4. The Symplectic, Metric, and Complex Structures of E-Phase Space

For details of the constructions in this and the next sections, see [13,14]. First, we shall establish some notation. To simplify, we shall write $\rho \left(x\right)=\rho_{x}$ and $\phi \left(x\right)=\phi_{x}$. A point (ρ,ϕ) in e-phase space $T^{*}S^{+}$ has coordinates $\left(\rho_{x},\phi_{x}\right)$. A curve in e-phase space parametrized by λ is the one-dimensional set of points (ρ(λ),ϕ(λ)). The vector $\bar{V}$ tangent to the curve is written

(31) $$\bar{V}=\frac{d}{d\lambda }=\int dx\left(\frac{d\rho_{x}}{d\lambda }\frac{\delta }{\delta \rho_{x}}+\frac{d\phi_{x}}{d\lambda }\frac{\delta }{\delta \phi_{x}}\right)=V^{\alpha x}\frac{\delta }{\delta X^{\alpha x}}$$

where $dx=d^{3N}x$, we introduced the discrete index α=1,2 to stand for ρ and ϕ, respectively,

(32) $$X^{1x}=\rho_{x}\;\mathrm{and}\;X^{2x}=\phi_{x},\;$$

and we adopt the convention of integration over repeated indices. The directional derivative of a functional F[ρ,ϕ] along $\bar{V}$ is

(33) $$\frac{dF}{d\lambda }=\int dx\left(\frac{\delta F}{\delta \rho_{x}}\frac{d\rho_{x}}{d\lambda }+\frac{\delta F}{\delta \phi_{x}}\frac{d\phi_{x}}{d\lambda }\right)=\frac{\delta F}{\delta X^{\alpha x}}\frac{dX^{\alpha x}}{d\lambda }=\tilde{\nabla }F\left[\bar{V}\right],$$

where $\tilde{\nabla }$ is the gradient in $T^{*}S^{+}$, that is,

(34) $$\tilde{\nabla }F=\int dx\left(\frac{\delta F}{\delta \rho_{x}}\tilde{\nabla }\rho_{x}+\frac{\delta F}{\delta \phi_{x}}\tilde{\nabla }\phi_{x}\right)=\frac{\delta F}{\delta X^{\alpha x}}\tilde{\nabla }X^{\alpha x},$$

where

(35) $$\tilde{\nabla }X^{1x}=\tilde{\nabla }\rho_{x}\;\mathrm{and}\;\tilde{\nabla }X^{2x}=\tilde{\nabla }\phi_{x}$$

are the basis covectors. (The tilde “~” serves to distinguish the gradient ∇ on $S^{+}$ from the gradient $\tilde{\nabla }$ on $T^{*}S^{+}$).

Once local coordinates $\rho_{x}$ and $\phi_{x}$ on the e-phase space have been identified, there is a natural symplectic form

(36) $$\Omega \left[\cdot ,\cdot \right]=\int dx\;\left(\tilde{\nabla }\rho_{x}\left[\cdot \right]⊗\tilde{\nabla }\phi_{x}\left[\cdot \right]-\tilde{\nabla }\phi_{x}\left[\cdot \right]⊗\tilde{\nabla }\rho_{x}\left[\cdot \right]\right),$$

where ⊗ is the tensor product. The action of Ω[·,·] on two vectors $\bar{V}=d/d\lambda$ and $\bar{U}=d/d\mu$ is given by

(37) $$\Omega \left[\bar{V},\bar{U}\right]=\int dx\;\left[V^{1x}U^{2x}-V^{2x}U^{1x}\right]=\Omega_{\alpha x,\beta x^{\prime }}V^{\alpha x}U^{\beta x^{\prime }},\;$$

where the components of Ω displayed as a matrix are

(38) $$\left[\Omega_{\alpha x,\beta x^{\prime }}\right]=\left[\begin{array}{cc} 0 & 1\\ -1 & 0 \end{array}\right]\delta_{xx^{\prime }},$$

and $\delta_{xx^{\prime }}=\delta \left(x,x^{\prime }\right)$ is the Dirac δ function.

Time evolution is required to reproduce the continuity equation. It will also preserve the normalization constraint,

(39) $$\tilde{N}=0\;\mathrm{where}\;\tilde{N}=1-\left|\rho \right|\;\mathrm{and}\;\left|\rho \right|\overset{\mathrm{def}}{=}\int dx\;\rho_{x}.$$

Indeed, one can check that

(40) $$\partial_{t}\tilde{N}=\left\{\tilde{N},\tilde{H}\right\}=0.$$

The embedding e-configuration space $S^{+}$ and e-phase space $T^{*}S^{+}$ are (without loss of generality [14]) assigned the simplest possible geometries; namely, they are flat. With this choice, the $T^{*}S^{+}$ metric is

(41) $$\delta {\tilde{\ell }}^{2}=\int dx\left[\frac{\hbar }{2\rho_{x}}{\left(\delta \rho_{x}\right)}^{2}+\frac{2\rho_{x}}{\hbar }\delta \phi_{x}^{2}\right]=G_{\alpha x,\beta x^{\prime }}\delta X^{\alpha x}\delta X^{\beta x^{\prime }}$$

where the metric tensor *G* displayed as a matrix is

(42) $$\left[G_{\alpha x,\beta x^{\prime }}\right]=\left[\begin{array}{cc} \frac{\hbar }{2\rho_{x}}\delta_{xx^{\prime }} & 0\\ 0 & \frac{2\rho_{x}}{\hbar }\delta_{xx^{\prime }} \end{array}\right],$$

where *ℏ* is just an arbitrary constant that will eventually be identified with Planck’s constant h/2π. It is interesting, however, that in ED the role of *ℏ* can be characterized geometrically. The constant *ℏ* determines the relative weights with which the coordinate $\delta \rho_{x}$ and the momentum components $\delta \phi_{x}$ contribute to the length of a vector $\left(\delta \rho_{x},\delta \phi_{x}\right)$.

The joint existence of symplectic Ω and metric *G* structures implies the existence of a complex structure described by a tensor *J* defined by

(43) $${J^{\alpha x}}_{\beta x^{\prime }}=-G^{\alpha x,\gamma x^{\prime \prime }}\Omega_{\gamma x^{\prime \prime },\beta x^{\prime }}\;\mathrm{or}\;\left[{J^{x}}_{x^{\prime }}\right]=\left[\begin{array}{cc} 0 & -\frac{2\rho_{x}}{\hbar }\delta_{xx^{\prime }}\\ \frac{\hbar }{2\rho_{x}}\delta_{xx^{\prime }} & 0 \end{array}\right].$$

This suggests a canonical transformation to complex coordinates,

(44) $$\psi_{x}=\rho_{x}^{1/2}e^{i\phi_{x}/\hbar },$$

with conjugate momenta $i\hbar \psi_{x}^{*}$ that are naturally adapted to the complex structure (see [13,14]). In wave function coordinates, Equations (41) and (42) become

(45) $$\delta {\tilde{\ell }}^{2}=2\hbar \int dx\;\delta \psi_{x}^{*}\delta \psi_{x}=2\hbar \langle \delta \psi |\delta \psi \rangle ,\;\left[G_{xx^{\prime }}\right]=-i\delta_{xx^{\prime }}\left[\begin{array}{cc} 0 & 1\\ 1 & 0 \end{array}\right].$$

Since the transformation (44) is canonical, the components of the symplectic tensor Ω, Equation (38), remain unchanged.

### 3.5. Hamilton–Killing Flows

We seek an Entropic Dynamics that preserves the natural geometric structures of the e-phase space. This requires a Hamiltonian $\tilde{H}$ that simultaneously preserves the symplectic structure Ω and the metric structure *G*. In order to generate flows that are simultaneously Hamilton flows and Killing flows, $\tilde{H}$ must satisfy

(46) $${£}_{H}\Omega =0\;\mathrm{and}\;{£}_{H}G=0,$$

where ${£}_{H}$ is the Lie derivative along the Hamiltonian vector field $\bar{H}$. As shown in [13,14], the Hamilton–Killing flows (or HK flows) that also preserve normalization are generated by Hamiltonian functionals that are bilinear in $\psi_{x}^{*}$ and $\psi_{x}$,

(47) $$\tilde{H}\left[\psi ,\psi^{*}\right]=\int dxdx^{\prime }\;\psi_{x}^{*}{\hat{H}}_{xx^{\prime }}\psi_{x^{\prime }},$$

which, incidentally, implies the linearity of the Schrödinger equation. In addition, we require that $\tilde{H}$ generate evolution in entropic time; that is, it must agree with Equation (28) in order to reproduce the continuity Equation (26). This constrains the integration constant $\tilde{F}\left[\rho \right]$ to a bilinear functional with a potential V(x) that is local in configuration space. The resulting Hamiltonian is

(48) $${\tilde{H}}_{\dot{\xi }}\left[\psi ,\psi^{*}\right]=\int dx\psi_{x}^{*}\sum_{n}\left(\frac{1}{2m_{n}}\delta^{ab}\left(\frac{\hbar }{i}\partial_{na}-m_{n}{\dot{\xi }}_{na}\right)\left(\frac{\hbar }{i}\partial_{nb}-m_{n}{\dot{\xi }}_{nb}\right)+V\left(x\right)\right)\psi_{x},$$

where we used

(49) $${\dot{\xi }}_{A}=m_{AC}{\dot{\xi }}^{C}=\sum_{n^{\prime }}m_{n}\delta_{nn^{\prime }}\delta_{ac}{\dot{\xi }}_{n}^{c}=m_{n}\delta_{ac}{\dot{\xi }}_{n}^{c}=m_{n}{\dot{\xi }}_{na}.$$

This bilinear Hamiltonian leads to a linear Schrödinger equation,

(50) $$i\hbar \partial_{t}\psi =\sum_{n}\frac{1}{2m_{n}}\delta^{ab}\left(\frac{\hbar }{i}\partial_{na}-m_{n}{\dot{\xi }}_{na}\right)\left(\frac{\hbar }{i}\partial_{nb}-m_{n}{\dot{\xi }}_{nb}\right)\psi +V\left(x\right)\psi .$$

Naturally, in order that linear and angular momentum be conserved, we shall further demand that the transformations ST1, Equation (1), which are a subgroup of ST2 and ST3, be a symmetry of the system. To achieve this, we require that the potential V(x) be a function of the interparticle distances,

(51) $$V\left(x\right)=V(\{|{\vec{x}}_{n}-{\vec{x}}_{m}|\}).$$

## 4. A Quantum Criterion for Best Matching

There are two foundational pillars of the ED approach to QM: one refers to kinematics, the other to dynamics. The former is the kinematic concept of Hamilton–Killing flows, Equation (46), based on the information metric and symplectic tensors. The latter is the explanation of the dynamics of probabilities as a form of entropic updating described in Section 3. It is only natural to expect that, in its ED version, the logic of quantum best matching (BM) will also involve the same two tensors *G* and Ω. Consider two states Ψ and *X*; in wave function coordinates,

(52) Ψμx=ψxiℏψx∗andXνy=χyiℏχy∗,

where the discrete index μ=1,2 stands for ψ and its momentum iℏψ, respectively. From Ω and *G*, Equations (38) and (45), we define the inner product (see [13,14]) of $\psi_{x}$ and $\chi_{x}$ by

(53) $$\frac{1}{2\hbar }\int dxdy\;\left(G_{\mu x,\nu y}+i\Omega_{\mu x,\nu y}\right)\Psi^{\mu x}X^{\nu y}=\int dx\;\psi_{x}^{*}\chi_{x}\overset{\mathrm{def}}{=}\langle \psi |\chi \rangle .$$

Our goal is to find the shift $x_{n}^{a}\to x_{n}^{a}+\xi_{n}^{a}$ so that the state $\psi_{t+dt}$ is best matched relative to the slightly earlier state $\psi_{t}$. The strategy is to minimize an appropriate measure of the mismatch between $\psi_{t}$ and $\psi_{t+dt}$. We assume that the states $\psi_{t}$ and $\psi_{t+dt}$ are normalized and that $\psi_{t+dt}$ is obtained by time-evolving $\psi_{t}$ using the Hamiltonian ${\tilde{H}}_{\dot{\xi }}$, Equations (47) and (48). We propose the following candidate for a measure of mismatch,

(54) $$\delta \left(\dot{\xi }\right)={\left|\langle \psi_{t}|\psi_{t+dt}\rangle -1\right|}^{2}.$$

Then, the BM shift ${\dot{\xi }}_{\mathrm{best}}$ is found by minimizing the measure $\delta (\dot{\xi })$. Other mismatch measures are in principle possible, but $\delta (\dot{\xi })$ recommends itself because **(a)** it involves geometric structures (Ω and *G*) that are natural to QM, **(b)** it obeys the natural limiting condition of vanishing as dt→0 so that $\psi_{t}$ is best matched relative to itself, **(c)** one can check that $\delta (\dot{\xi })$ does have a minimum and, most importantly, **(d)** $\delta (\dot{\xi })$ is useful and convenient in actual practice. Equation (54) can be written in a more convenient form using

(55) $$|\psi_{t+dt}\rangle \;=\;|\psi_{t}\rangle \;+\;|\delta \psi_{t}\rangle \;=\;|\psi_{t}\rangle \;+\;\frac{dt}{i\hbar }{\hat{H}}_{\dot{\xi }}|\psi_{t}\rangle .$$

Then, we find

(56) $$\delta \left(\dot{\xi }\right)\;=\;|\langle \psi_{t}|\delta \psi_{t}\rangle {|}^{2}=\frac{dt^{2}}{\hbar^{2}}\langle \psi_{t}|{\hat{H}}_{\dot{\xi }}{|\psi_{t}\rangle }^{2},$$

or,

(57) $$\delta \left(\dot{\xi }\right)=\frac{dt^{2}}{\hbar^{2}}{\tilde{H}}_{\dot{\xi }}^{2}.$$

Next, we analyze the consequences of minimizing the mismatch $\delta (\dot{\xi })$.

### 4.1. Best Matching Under Rigid Translations

In this section, we formulate a QM model that is relational with respect to rigid translations. Minimizing (57) with ${\tilde{H}}_{\dot{\xi }}$ given by (48) and ${\dot{\xi }}^{a}={\dot{\lambda }}^{a}$,

(58) $$\frac{\partial }{\partial {\dot{\lambda }}^{a}}{\tilde{H}}_{\dot{\lambda }}=0,$$

yields

(59) $$0=-\int dx\psi^{*}\sum_{n}\delta^{ab}\left(\frac{\hbar }{i}\partial_{nb}-m_{n}{\dot{\lambda }}_{b}\right)\psi =M{\dot{\lambda }}^{a}-\delta^{ab}\int dx\psi^{*}\sum_{n}\frac{\hbar }{i}\partial_{na}\psi ,$$

where $M=\sum_{n}m_{n}$. Therefore, the optimal shift is given by

(60) $$M{\dot{\lambda }}_{a}=\langle \psi |{\hat{P}}_{a}|\psi \rangle ={\tilde{P}}_{a},$$

where the (expected) total momentum,

(61) $${\tilde{P}}_{a}\left[\psi ,\psi^{*}\right]=\int dx\psi^{*}\sum_{n}\frac{\hbar }{i}\partial_{na}\psi ,$$

is the generator of global translations. The interpretation is straightforward. If we are given a sequence of consecutive states, the shift velocity ${\dot{\lambda }}^{a}$ that achieves equilocality is given by the expected velocity of the center of mass.

Conversely, to formulate a relational dynamics with evolution given by (48) with ${\dot{\lambda }}^{a}={\dot{\xi }}^{a}$ chosen to achieve best matching, we must impose that ${\dot{\lambda }}^{a}$ be a constant in time and require that solutions be constrained to the subspace of the full Hilbert space with a given total momentum,

(62) $${\tilde{P}}_{a}\left[\psi ,\psi^{*}\right]-M{\dot{\lambda }}_{a}=0\;\mathrm{or}\;\langle \psi |\left(\hat{P}-M{\dot{\lambda }}^{a}\right)|\psi \rangle =0.$$

This completes our formulation of an ED model that is relational relative to the rigid translations ST2, but we can can go a bit further.

The Galilean transformations ST4, Equation (4), allow us to describe the system in the center of mass frame, that is, ${\dot{\lambda }}^{a}=0$, which means that every state is best matched relative to the previous one and spatial points with the same $\vec{x}$ coordinates are equilocal. The corresponding Hamiltonian, Equation (48), is

(63) $${\tilde{H}}_{0}\left[\psi ,\psi^{*}\right]=\int dx\psi_{x}^{*}\sum_{n}\left(\frac{-\hbar^{2}}{2m_{n}}\delta^{ab}\partial_{na}\partial_{nb}+V(\{|{\vec{x}}_{n}-{\vec{x}}_{m}\left|\}\right)\right)\psi_{x},$$

and quantum states ψ are restricted to the subspace constrained by

(64) $${\tilde{P}}_{a}\left[\psi ,\psi^{*}\right]=0\;\mathrm{or}\;\langle \psi |\hat{P}|\psi \rangle =0.$$

We conclude by noting that BM imposes expected value constraints. In the standard approach to quantizing theories with constraints, questions arise as to whether the constraints of the classical theory should, after quantization, be imposed on the ontic microstates, on the operators, on the quantum states, or on expectation values. The ED approach to BM provides a crisp answer: the quantum constraints that express relationality are to be imposed on expectation values.

### 4.2. Best Matching Under Rigid Translations and Rotations

In this section, we formulate a QM model that is relational with respect to the ST3 transformations of both rigid translations and rotations, ${\dot{\xi }}_{n}^{a}=\varepsilon^{abc}{\dot{\zeta }}_{b}x_{nc}+{\dot{\lambda }}^{a}$. For simplicity, we shall assume that relationality with respect to translations has already been imposed; the system is described in the center of mass frame, ${\dot{\lambda }}^{a}=0$, and quantum states are constrained by (64). Then, ${\dot{\xi }}_{n}^{a}=\varepsilon^{abc}{\dot{\zeta }}_{b}x_{nc}$ and the best matching condition (54) is written as

(65) $$\frac{\partial }{\partial {\dot{\zeta }}_{a}}{\tilde{H}}_{\dot{\xi }}=0.$$

Substituting ${\tilde{H}}_{\dot{\xi }}$ from by (48), after some straightforward algebra, we find

$$\frac{\partial {\tilde{H}}_{\dot{\xi }}}{\partial {\dot{\zeta }}_{a}}=-\int dx\psi^{*}\sum_{n}\varepsilon^{abc}x_{nb}\left(\frac{\hbar }{i}\partial_{nc}\right)\psi -\int dx\psi^{*}\sum_{n}m_{n}\varepsilon^{abd}{\dot{\xi }}_{nb}x_{nd}\psi .$$

The first integral on the right is recognized as the (expected) total angular momentum,

(66) $$1\mathrm{st}=-\int dx\psi^{*}\sum_{n}\varepsilon^{abc}x_{nb}\left(\frac{\hbar }{i}\partial_{nc}\right)\psi =-\langle \psi |{\hat{L}}^{a}|\psi \rangle =-{\tilde{L}}^{a}.$$

Substituting ${\dot{\xi }}_{n}^{a}=\varepsilon^{abc}{\dot{\zeta }}_{b}x_{nc}$ into the second integral,

(67) $$2\mathrm{nd}=-\int dx\psi^{*}\sum_{n}m_{n}\varepsilon^{abc}{\dot{\xi }}_{nb}x_{n}^{c}\psi =\int dx\psi^{*}\sum_{n}m_{n}\varepsilon^{bac}\varepsilon^{bde}{\dot{\zeta }}^{d}x_{n}^{e}x_{n}^{c}\psi$$

and using the identity $\varepsilon^{bac}\varepsilon^{bde}=\delta^{ad}\delta^{ce}-\delta^{ae}\delta^{cd}$, we find

(68) $$2\mathrm{nd}=\int dx\psi^{*}\sum_{n}m_{n}\left(\delta^{ab}x_{n}^{c}x_{n}^{c}-x_{n}^{a}x_{n}^{b}\right)\psi {\dot{\zeta }}_{b}=\langle \psi |{\hat{I}}^{ab}|\psi \rangle {\dot{\zeta }}_{b}={\tilde{I}}^{ab}\left[\psi ,\psi^{*}\right]{\dot{\zeta }}_{b},$$

where ${\hat{I}}^{ab}$ is recognized as the moment of inertia tensor and the functional ${\tilde{I}}^{ab}\left[\psi ,\psi^{*}\right]$ is its expected value. Combining (66) and (68), we find

(69) $$\frac{\partial {\tilde{H}}_{\dot{\xi }}}{\partial {\dot{\zeta }}_{a}}={\tilde{I}}^{ab}{\dot{\zeta }}_{b}-{\tilde{L}}^{a}=0.$$

The interpretation is that if we are given a sequence of consecutive states, the shift angular velocity ${\dot{\zeta }}^{a}$ that achieves minimal mismatch with respect to rotations is given by the expected angular velocity of the system,

(70) $${\dot{\zeta }}^{a}={\left({\tilde{I}}^{-1}\right)}_{b}^{a}{\tilde{L}}^{b}.$$

Thus, in the center of mass frame (${\tilde{P}}^{a}=0$) rotational relationality is implemented through a BM constraint that restricts solutions to the subspace of the full Hilbert space with a conserved expected total momentum given by

(71) $${\tilde{L}}^{a}={\tilde{I}}^{ab}{\dot{\zeta }}_{b}.$$

In conclusion, to formulate a relational dynamics with evolution given by (48) with ${\dot{\xi }}_{n}^{a}=\varepsilon^{abc}{\dot{\zeta }}_{b}x_{nc}+{\dot{\lambda }}^{a}$ chosen to achieve best matching, then we must require that solutions be constrained to the subspace of the full Hilbert space with total momentum and total angular momentum given by

(72) $$\langle \psi |\left(\hat{P}-M{\dot{\lambda }}^{a}\right)|\psi \rangle =0\;\mathrm{and}\;\langle \psi |\left({\hat{L}}^{a}-{\hat{I}}^{ab}{\dot{\zeta }}_{b}\right)|\psi \rangle =0.$$

This completes our formulation of an ED model that is relational relative to the rigid translations and rotations ST3.

So far, the situation seems closely analogous to the case of translations but in fact it is substantially different. While momentum conservation implies that the center of mass velocity ${\dot{\lambda }}^{a}$ must be constant in time, the conservation of angular momentum does not imply that the angular velocity ${\dot{\zeta }}^{a}$ is constant because the system is not a rigid body and the moment of inertia ${\tilde{I}}^{ab}=\langle \psi |{\hat{I}}^{ab}|\psi \rangle$ is not constant in time. Obvious exceptions are those states for which ${\tilde{L}}^{a}=0$. Furthermore, if we want a dynamics that is relational with respect to rotations then we ought to be able to transform to a rotating frame, but this is not a symmetry, at least not obviously so: one cannot just transform to a rotating frame and not expect the appearance of centrifugal forces. We meet here the quantum analogue of Newton’s bucket. The elegant way out of this quandary has been known for a long time. The resolution is via Einstein’s equivalence principle: one can formulate a dynamics that is relational with respect to rotations provided the centrifugal forces are interpreted as radial gravitational forces. From this broadened perspective, what is rotationally relational is not the system of particles by themselves, but the composite system of particles plus the gravitational field. This topic obviously deserves a more detailed study but its pursuit would take us beyond the more limited goals of this paper. Its further exploration will be taken up elsewhere.

## 5. Formal Developments

### 5.1. Action Principle

In the ED framework, the introduction of an action principle is not fundamental; it is a merely convenient way to summarize the content of an already established formalism. The usual procedure is reversed: given the Schrödinger Equation (50) and the desired constraints, one designs an action that reproduces them.

For simplicity, here we shall limit ourselves to the ST2 model and just consider relationality with respect to rigid translations. The generalization to include rotations is immediate. The starting point is to define the differential

(73) $$\delta A=\int_{t_{1}}^{t_{2}}dt\;\int_{R}dx\;\left[\delta \psi^{*}\left(i\hbar \partial_{t}\psi -{\hat{H}}_{\dot{\lambda }}\;\psi \right)-\left(i\hbar \partial_{t}\psi^{*}+{\hat{H}}_{\dot{\lambda }}^{*}\psi^{*}\right)\delta \psi \right]$$

where ${\tilde{H}}_{\dot{\lambda }}$ is given in (48) and the field variations δψ and $\delta \psi^{*}$ vanish at the boundary of the region *R*. Then, one integrates δA to obtain the action

(74) $$A=\int_{t_{1}}^{t_{2}}dt\;\left(\int_{R}dx\;i\hbar \psi^{*}\partial_{t}\psi -{\tilde{H}}_{\dot{\lambda }}\left(\;\psi ,\psi^{*}\right)\right).$$

Remarkably, it is not necessary to append additional terms to the action to enforce constraints. Varying with respect to ${\dot{\lambda }}_{a}\left(t\right)$ already gives the desired BM constraint, Equation (62). In the action formalism, the mismatch correction ${\dot{\lambda }}_{a}$ plays the role of a Lagrange multiplier.

### 5.2. Parametrized Entropic Dynamics

We have emphasized that the ED models developed here are already temporally relational in the sense that there are neither external clocks nor an external time. Our final goal is to make this temporal relationality explicit by producing a model in which entropic time *t* no longer plays the role of time but, instead, becomes a dynamical variable on a par with $\psi_{x}$.

The technique is well known from the canonical formalism of general relativity [21,22]. It is called “parametrization”, and the resulting theories are said to be “generally covariant”. The idea is to introduce an unphysical label $x^{0}$ that retains one of the important roles of time, namely, that of keeping track of the temporal ordering of states. Let

(75) $$t=t\left(x^{0}\right)\;\mathrm{and}\;\mathrm{let}\;\partial_{0}t=\frac{\partial t}{\partial x^{0}}\;$$

be the velocity of entropic time *t* with respect to the “time” label $x^{0}$. Then, $\psi (x,t\left(x^{0}\right))$ and $\dot{\lambda }\left(t\left(x^{0}\right)\right)$ become functions of $x^{0}$ which we will just write as $\psi (x,x^{0})$ and $\dot{\lambda }\left(x^{0}\right)$. The action (74) becomes

(76) $$A=\int_{t_{1}}^{t_{2}}dx^{0}\;\left(\int_{R}dx\;i\hbar \psi^{*}\partial_{0}\psi -\partial_{0}t\;{\tilde{H}}_{\dot{\lambda }}\left(\;\psi ,\psi^{*}\right)\right).$$

Note that A is linear in both the ψ velocity and the *t* velocity. This suggests that we can reinterpret *t* as one of the canonical coordinates provided we simultaneously reinterpret $-{\tilde{H}}_{\dot{\lambda }}\left(\;\psi ,\psi^{*}\right)=\pi_{0}$ as its conjugate momentum. The new action,

(77) $$A=\int_{t_{1}}^{t_{2}}dx^{0}\;\left(\pi_{0}\partial_{0}t+\int_{R}dx\;i\hbar \psi^{*}\partial_{0}\psi \right),$$

is in standard $\int p\dot{q}$ form. The canonical variables $\left\{t,\pi_{0},\psi ,i\hbar \psi^{*}\right\}$ cannot, however, be varied independently because they are subject to the so-called super-Hamiltonian constraint,

(78) $$\tilde{H}\overset{\mathrm{def}}{=}\pi_{0}+{\tilde{H}}_{\dot{\lambda }}\left(\;\psi ,\psi^{*}\right)=0,$$

which must, therefore, be appended to the action with its corresponding Lagrange multiplier,

(79) $$A=\int_{t_{1}}^{t_{2}}dx^{0}\;\left(\pi_{0}\partial_{0}t+\int_{R}dx\;i\hbar \psi^{*}\partial_{0}\psi -\beta \tilde{H}\right).$$

Now, we can vary all $\left\{t,\pi_{0},\psi ,i\hbar \psi^{*}\right\}$ independently and we can proceed to obtain equations of motion where $\beta \tilde{H}$ plays the role of a Hamiltonian. In particular,

(80) $$\partial_{0}t=\frac{\partial }{\partial \pi_{0}}\beta \tilde{H}=\beta ,$$

allows us to interpret β as the rate of change of entropic time *t* with respect to the label $x^{0}$, a quantity usually called the lapse function [2].

Constraints often generate invariances and the super-Hamiltonian $\tilde{H}$ is no different. An important consequence of including *t* among the dynamical variables is that the action (79) turns out to be invariant under the relabeling

(81) $$x^{0}\to {\bar{x}}^{0}={\bar{x}}^{0}\left(x^{0}\right).$$

The proof is immediate; just note that

(82) $$dx^{0}\;\beta =dx^{0}\partial_{0}t=d{\bar{x}}^{0}\partial_{\bar{0}}t=d{\bar{x}}^{0}\;\bar{\beta }.$$

The invariance with respect to the continuous gauge group (81) is referred to as general covariance.

To highlight the potential significance of the results above, we conclude with the observation that in the canonical quantization of gravity the super-Hamiltonian of the classical theory, $H_{\mathrm{class}}$, is constrained to vanish weakly (i.e., vanish on the manifold of solutions of the equations of motion) and this gives rise to the famous problem of time (see e.g., [17]). The problem is that upon quantization the quantum version of the constraint takes the form $\hat{H}\Psi =0$; the quantum Hamiltonian annihilates the physical quantum states. The Wheeler–DeWitt equation—the analogue of the Schrödinger equation—then implies that physical states cannot evolve.

Buckets of effort and ingenuity have been poured on this issue (see e.g., [18]) and we shall not muddy it further. Our sole concern here is to point out that ED evades all these problems because it constructs the quantum theory directly without first formulating a classical theory that must then be massaged according to quantization rules that to this day elicit controversy.

## 6. Conclusions

The ED approach to QM has been extended to formulate non-relativistic quantum models that are spatially relational with respect to rigid translations and rotations. Although only partially relational—absolute structures of simultaneity and Euclidean geometry are retained—our models still provide a useful testing ground for ideas that will prove useful in the context of more realistic relativistic theories.

The fact that within ED the positions of particles are meant to be ontic with definite values at all times, just as in classical mechanics, has allowed us to adapt and adopt some intuitions from Barbour and Bertotti’s classical framework. Nevertheless, the two frameworks are very different. Their classical measure of mismatch compares ontic particle configurations and is based on Jacobi’s action. In contrast, our quantum measure of mismatch compares epistemic quantum states and is adapted to the metric and symplectic structures of the epistemic phase space.

The case of a single particle serves to illustrate the difference. While it makes no sense to consider the relational classical motion of a single particle, in the quantum case a relational dynamics makes sense even for a single particle because what are being best matched are infinite dimensional wave functions.

We have shown that even in its previous formulations ED was already temporally relational. Here, we made this feature explicit and, as an example of a development that might prove valuable in the context of quantum gravity, the non-relativistic quantum model was rewritten in generally covariant form. This toy model shows that the ED approach evades the analogue of what in quantum gravity has been called the problem of time.

Finally, we note that the relational ED framework developed here can be applied beyond the example of particles. It is expected to apply to any model with redundancy in description, and this potentially includes all fundamental theories such as electromagnetism, Yang–Mills theories and, possibly, gravity. 

## Data Availability

The original contributions presented in this study are included in the article. Further inquiries can be directed to the corresponding authors.

## Abbreviations

The following abbreviations are used in this manuscript:

ED	Entropic Dynamics
QM	Quantum Mechanics
BM	Best Matching
